# Ned-19 inhibition of parasite growth and multiplication suggests a role for NAADP mediated signalling in the asexual development of *Plasmodium falciparum*

**DOI:** 10.1186/s12936-017-2013-7

**Published:** 2017-09-12

**Authors:** Pablo Suárez-Cortés, Guido Gambara, Annarita Favia, Fioretta Palombi, Pietro Alano, Antonio Filippini

**Affiliations:** 1 Dipartimento di Malattie Infettive, Istituto Superiore di Sanità, Viale Regina Elena n. 299, 00161 Rome, Italy; 2 Department of Vector Biology, Max-Planck Institute for Infection Biology, Charitéplatz 1, 10117 Berlin, Germany; 3 Department of Anatomy, Histology, Forensic Medicine and Orthopedics, Section of Histology and Medical Embryology, Sapienza University of Rome, Rome, Italy; 4 Nucleic Acids Laboratory, Institute of Molecular Biology and Pathology, National Research Council (IBPM-CNR), Department of Biology and Biotechnologies, Sapienza University, Rome, Italy

**Keywords:** Ned-19, NAADP, Malaria, Calcium signalling, *Plasmodium falciparum*, NAADP receptor, Antimalarial drugs, *P. falciparum* development

## Abstract

**Background:**

Although malaria is a preventable and curable human disease, millions of people risk to be infected by the *Plasmodium* parasites and to develop this illness. Therefore, there is an urgent need to identify new anti-malarial drugs. Ca^2+^ signalling regulates different processes in the life cycle of *Plasmodium falciparum*, representing a suitable target for the development of new drugs.

**Results:**

This study investigated for the first time the effect of a highly specific inhibitor of nicotinic acid adenine dinucleotide phosphate (NAADP)-induced Ca^2+^ release (Ned-19) on *P. falciparum*, revealing the inhibitory effect of this compound on the blood stage development of this parasite. Ned-19 inhibits both the transition of the parasite from the early to the late trophozoite stage and the ability of the late trophozoite to develop to the multinucleated schizont stage. In addition, Ned-19 affects spontaneous intracellular Ca^2+^ oscillations in ring and trophozoite stage parasites, suggesting that the observed inhibitory effects may be associated to regulation of intracellular Ca^2+^ levels.

**Conclusions:**

This study highlights the inhibitory effect of Ned-19 on progression of the asexual life cycle of *P. falciparum*. The observation that Ned-19 inhibits spontaneous Ca^2+^ oscillations suggests a potential role of NAADP in regulating Ca^2+^ signalling of *P. falciparum*.

**Electronic supplementary material:**

The online version of this article (doi:10.1186/s12936-017-2013-7) contains supplementary material, which is available to authorized users.

## Background

With 3.3 billion of people at risk to be infected and an estimated death toll of 429,000 in 2015 [1] malaria remains a great threat in tropical countries. Artemisinin combination therapies, although highly effective, have met with the development of parasite resistance in recent years, exacerbating the need for the identification of new anti-malarial drugs [2].

In *Plasmodium falciparum*, Ca^2+^ signalling regulates different physiological processes involved in the life cycle of parasite, such as erythrocyte invasion [3, 4], merozoite egress from the infected erythrocyte [5] and parasite development [6]. As well as in higher eukaryotic cells, different Ca^2+^ stores have been found in *Plasmodium*: endoplasmic reticulum, acidocalcisomes, mitochondria and the digestive vacuole. Acidocalcisomes are acidic organelles rich in Ca^2+^ and other cations bound to phosphate polymers. A Ca^2+^-ATPase, a bafilomycin-sensitive vacuolar V-H^+^-ATPase, a V-H^+^-PPase and a Ca^2+^–H^+^ antiporter localize on the membrane of these organelles, regulating Ca^2+^ balance [7]. It has been proposed that another acidic organelle of *P. falciparum*, the food vacuole, could be a Ca^2+^ store sensitive to thapsigargin, bafilomycin and NH_4_Cl [8]. Ca^2+^ homeostasis in *Plasmodium* is mainly regulated by two Ca^2+^-ATPase, the thapsigargin-insensitive PfATP4 [9] and the thapsigargin-sensitive sarco/endoplasmic reticulum Ca^2+^-ATPase (SERCA) orthologue PfATP6 [10]. Scheibel and co-workers showed that both Ca^2+^ and calmodulin antagonists inhibit the growth of *P. falciparum* [11]. Moreover, the identification of SERCA as a target of artemisinins [10] highlights the crucial role of Ca^2+^ signalling in the life cycle of the parasite.

In higher eukaryotic cells, different intracellular second messengers finely regulate the spatio-temporal fluctuation of cytosolic Ca^2+^ concentration, mobilizing calcium from different intracellular stores. Inositol 1,4,5-bisphosphate (IP3) [12], cyclic ADP-ribose (cADPR) [13] and nicotinic acid adenine dinucleotide phosphate (NAADP) [14] have been so far identified as Ca^2+^-mobilizing second messengers in higher eukaryotic cells. The second messenger NAADP was described for the first time as a potent Ca^2+^ mobilizing agent in sea urchin eggs [14] and subsequently in ascidian and starfish oocytes [15, 16], in plants [17] and in higher eukaryotic cells [18–21], suggesting a highly conserved feature in evolution for this molecule.

In *Apicomplexa*, second messengers involved in Ca^2+^ mobilization from intracellular organelles have been poorly investigated. At present only a few works have investigated the role of IP3 and cADPR in Ca^2+^ release in *P. falciparum* [22–24] and none focused on NAADP, possibly for the absence in the parasite genome of sequences homologous to the putative two pore channels (TPCs) NAADP-receptor. Recently, Ned-19 has been identified by virtual screening as a specific inhibitor of NAADP-induced calcium signalling in sea urchin eggs and pancreatic beta cells, and was shown to bind NAADP receptors as a fluorescent probe [25]. Subsequently, Ned-19 mediated Ca^2+^ signalling inhibition and its biological effects have been reported in different mammalian cells. Ned-19 has been shown to inhibit histamine-induced secretion of von Willebrand factor (vWF) in endothelial cells [20], endothelin-1 (ET-1)-induced contraction of smooth muscle cells [26], NAADP-induced acrosome reaction in mammalian spermatozoa [27] and exocytosis of cytolytic granules in cytotoxic T Lymphocytes and VEGF-induced neoangiogenesis [28, 29].

The aim of this study was to investigate the effect of Ned-19 on the blood stage development of *P. falciparum*. Results show for the first time that Ned-19 specifically impairs both the growth and the ability to transform into multinucleate schizonts of the asexual trophozoite stages, suggesting a crucial role of NAADP in life cycle progression. This work also shows that Ned-19 inhibits spontaneous Ca^2+^ oscillations in early ring and trophozoite stages, which also suggests an important role of NAADP in Ca^2+^ homeostasis of *P. falciparum*.

## Methods

### *Plasmodium falciparum* parasites and cultures

Parasites from clone 3D7 [30] were cultured in 0^+^ human red blood cells at 5% haematocrit in RPMI 1640 plus hypoxanthine 50 mg/mL, HEPES 25 mM, 0.225% sodium bicarbonate and 10 mg/mL gentamicin, supplemented with 10% heat inactivated human serum. Parasites were kept at 37 °C, in a 2% O_2_, 5% CO_2_ and 93% N_2_ atmosphere. Percoll cushion and sorbitol treatment for parasite synchronization were performed as described [31, 32] previously. In parasite synchronization, sorbitol treatment of newly invaded parasites from Percoll purified schizonts was performed 3 h after Percoll treatment to obtain a parasite synchronization window of maximum 3 h. For gametocyte production, asynchronous parasites were grown to high parasitaemia (>8%) and culture medium was doubled at this point. The day after, 50 mM N-acetylglucosamine was added to medium and maintained for 3 days, until no asexual parasites were detected in the culture. Stage II gametocytes were detected 48 h after the addition of N-acetylglucosamine, while mature stage V appeared from 9 days after the treatment.

Parasitaemia was measured through Giemsa staining of culture blood smears (counting of at least 2000 RBCs) or FACS using CYBRGreen staining as previously described (counting of at least 50,000 cells) [33]. FACSAria I (BD Biosciences, Erembodegem, Belgium) equipped with three lasers (488, 635 and 407 nm violet solid state laser) was used to determine parasitaemia to a precision of 0.1%. The results were analyzed by BD FACSDiva Software version 6.1.3 (BD Biosciences).

### Ned-19 and Ned-20 treatments

Ned-19 (*Tocris* bioscience) was resuspended in a stock solution of sterile dimethyl sulfoxide (DMSO) at 100 mM and kept at −20 °C until added to the *Plasmodium* cultures at the specified concentrations. In the case of Ned-20 the stock solution was kindly provided by Grant Churchill (Oxford University) at 10 mM and kept at −20 °C until use. Control cultures were incubated with a DMSO concentration equivalent to that of the treated cultures. Differences in parasitaemia between treated and untreated cultures were evaluated through Student’s t test.

### Microscopy

Parasite cultures were incubated with 200 μM Ned-19 and 1 μM Lysotracker Green DND-26 (ThermoFisher Scientific) for 30 min at 37 °C in agitation and observed with a fluorescence microscope. A Zeiss Observer.Z1 inverted microscope was used to visualize live samples. Images were acquired using a Zeiss AxioCam MRm Rev. 3 FireWire camera through a Zeiss C-Apochromat 63x/1, 20 objective. Filters used to detect fluorescence were EX: 365–395, EM: 445–450 (Ned-19) and EX: 440–470, EM: 525–550 (Lysotracker Green DND-26). Giemsa-stained smears were examined to confirm stages of the synchronized parasites: proportions >90% of trophozoites, schizonts and ring forms were, respectively, observed at 32, 46 and 48 h after synchronization.

### Electron microscopy

Parasite culture samples enriched in infected red blood cells by MACS (Magnetic-activated cell sorting) were fixed overnight in cold 2.5% glutaraldehyde in 0.1 M cacodylate buffer, postfixed in 2% osmium tetroxide for 2 h and treated for 30 min with 1% tannic acid in 0.05 M cacodylate buffer. Pellets were then dehydrated in ethanol and processed for Epon embedding. Ultrathin sections were contrasted in lead hydroxide and analyzed in a Hitachi 7000 transmission electron microscope.

### Calcium imaging

In Ca^2+^ imaging experiments, protocol was adapted from [6], using the ratiometric Fura-2-AM as calcium reporter. Samples from cultures at high parasitaemia (5–10%) of the desired parasite stage were generated through parasite synchronization as follows: for early rings, parasites were harvested 50 h after the initial Percoll treatment, while for early trophozoites parasites were harvested 26 h after Percoll treatment. Cultures where then washed in BSA− medium for Ca^2+^ imaging (RPMI 1640 medium without phenol red supplemented with 25 mM HEPES, 24 mM sodium bicarbonate, 0.5 g/L l-glutamine and 50 mg/L hypoxanthine) and resuspended at 5% haematocrit in loading medium [BSA− medium supplemented with 1:100 PowerLoad (ThermoFisher Scientific)] with 3 μM Fura-2-AM (ThermoFisher Scientific) at 37 °C for 150 min in agitation. Loading medium was then washed away and cells were resuspended at 2.5% haematocrit in BSA+ medium [BSA− medium supplemented with 0.5% Albumax I and 25 mg/mL gentamicin (Sigma)]. Ned-19 100 μM or 0.1% DMSO was added to 1 mL of this cell suspension. This was plated in glass-bottomed 30 mm dishes previously coated with poly-l-lysine and incubated in a 2% O_2_, 5% CO_2_ and 93% N_2_ atmosphere for 45 min. Unbound cells were washed by gently rinsing the surface of the plate and substituting supernatant with BSA+ medium with Ned-19 or DMSO. Medium volume was then adjusted to 1 mL of BSA+ with Ned-19 or DMSO. This treatment resulted in cells treated with Ned-19 or DMSO for at least 45 min prior to the beginning of measurements, and the Ned-19 and DMSO treatments being kept during calcium imaging.

Ca^2+^ mobilization was measured in presence or absence of Ned-19. Plates with Fura-2-loaded cells were placed into a culture chamber at 37 °C on the stage of an inverted fluorescence microscope (Nikon, TE2000E), connected to a cooled CCD camera (512B Cascade, Princeton Instruments, AZ). Samples were illuminated alternately at 340 and 380 nm using a random access monochromator (Photon Technology International, NJ) and emission was detected using a 510 nm emission filter. Images (1 set of emission at 340 and 380 nm every 1.5 s) were acquired using Metafluor software (Universal Imaging Corporation, Downingtown, PA).

Images were analysed by generating square 4 × 4 mm ROIs (region of interest) encompassing a single parasite and recording the intensity of the emission for the ROI at 340 and 380 nm. Background was normalized for each ROI using a ROI the size of the full image, and ratio (R) was calculated as F_(340)_/F_(380)_ for each ROI and time-point, obtaining time-courses for each ROI. R values were then normalized as R/R_min_−1.

OCTAVE free software with the script findpeaksfit.m by T.C. O’Haver [34] was used to identify and measure height of peaks, given as R/R_min_−1 values. Oscillation series were subdivided into fragments of 100 s each and script was run with settings as follows: x, y, SlopeThreshold = 0.0005, AmpThreshold = 0, smoothwide = 3, peakgroup = 3, smoothtype = 3, peakshape = 1, extra = 0, NumTrials = 0, autozero = 1, fixedparameters = 0, plots = 0. Differences between the height of peaks in DMSO and Ned-19 treated parasites were evaluated through unpaired Student’s t test analysis. For DMSO treated rings, N (number of time-courses) = 6, n (total number of peaks detected) = 622. For Ned-19 treated rings, N = 6, n = 265. In the case of trophozoites, for DMSO treated parasites N = 8, n = 349, for Ned-19 treated parasites N = 3, n = 150.

## Results

### Ned-19 inhibits the asexual development of *Plasmodium falciparum*

**Fig. 1:**
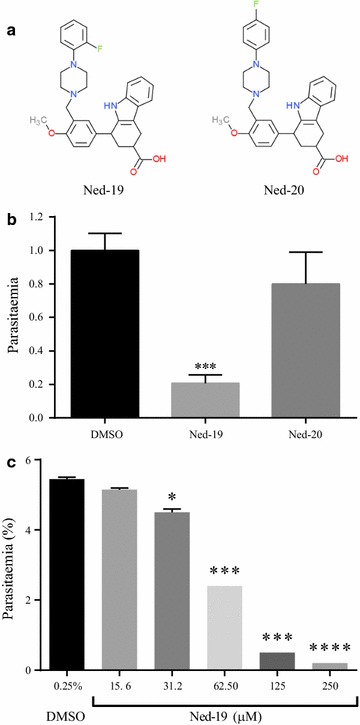
Ned-19 specific blockage of *P. falciparum* asexual growth. **a** Structure of Ned-19 and of its inactive analogue Ned-20, adapted from Rosen et al. **b** Synchronous early ring stage parasites (initial parasitaemia 0.16%) were cultured for 48 h in the presence of 100 μM concentration of the indicated compounds. At the end of the incubation, parasitaemias were measured through Giemsa-stained preparations. Parasitaemia of the DMSO treated culture (1.08%) was set as 1. N = 3. ***p < 0.001. *Error bar* SEM. **c** Late asexual parasites (24 h post invasion, initial parasitaemia 1%) were cultured with the indicated concentrations of Ned-19 for 24 h. Parasitaemia was then measured through FACS counting of at least 50.000 cells per sample. N = 2. *p < 0.05; ***p < 0.001; ****p < 0.0001. *Error bar* range/2

To determine whether the highly selective NAADP antagonist Ned-19 affected the asexual cycle of *P. falciparum*, the effects of this compound and of its inactive analogue Ned-20, unable to inhibit NAADP-mediated Ca^2+^ release in other organisms [35], were evaluated on synchronized early asexual stages of parasite clone 3D7, which were incubated for 48 h in the presence of Ned-19, Ned-20 or DMSO (100 μM). The parasitaemia of the culture incubated with Ned-19 showed a fivefold reduction compared to the DMSO-treated parasites. In contrast, the Ned-20 treated culture did not show a significant reduction in parasitaemia, supporting the specificity of Ned-19 in impairing the parasite asexual growth (Fig. 1b). Additional experiments showed that Ned-19 impairs parasite growth in a dose-dependent manner (Fig. 1c).

### Ned-19 blocks development of early trophozoites and prevents schizont maturation

**Fig. 2:**
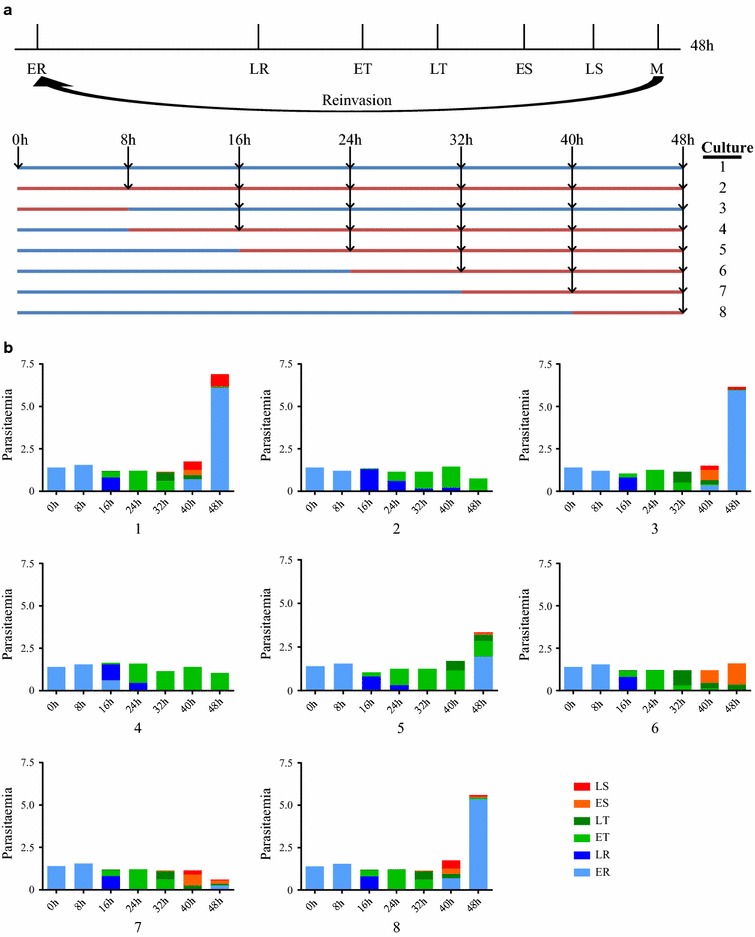
Ned-19 activity on different stages of the *P. falciparum* asexual cycle. Synchronized ring stage parasites (initial parasitaemia 1.4%) were used to yield subcultures, numbered 1–8, which were incubated with DMSO control (*blue line*) or exposed to 100 μM Ned-19 (*red line*) at different times of the parasite asexual cycle. **a** Stages of the parasite asexual cycle. *Black arrows* indicate times at which the cultures were sampled. **b** *Histograms* showing parasitaemia and distribution of the different parasite stages for the cultures in each time-point. *ER* early rings, *LR* late rings, *ET* early trophozoites, *LT* late trophozoites, *ES* early schizonts, *LS* late schizonts. A representative sample of these forms is shown in Additional file 2: Figure S2

To pinpoint the stage(s) of the parasite life cycle affected by Ned-19, a time course spanning one parasite asexual cycle was performed. A tightly synchronized culture of newly invaded ring stage parasites (synchronization window of 3 h) was produced and treated as described in Fig. 2. A control subculture was exposed to DMSO (subculture 1), one was treated with 100 μM Ned-19 for the entire cycle (subculture 2), one for the initial 8 h only (subculture 3), and in five additional subcultures (4–8) Ned-19 was added at 8 h intervals, at 8, 16, 24, 32 and 40 h of the parasite asexual development.

Parasite numbers and morphology were determined in Giemsa stained smears at all time points of all subcultures. Results indicated that presence of Ned-19 limited to the first 8 h after parasite invasion, at the very early ring stage parasites (subculture 3), or after 40 h, at the late schizont stage (subculture 8), did not affect the completion of the life cycle and the invasion of new red blood cells by the merozoites produced in schizogony. In contrast, parasite development was affected if Ned-19 was present between 8 and 40 h post-invasion. Timing and length of Ned-19 exposure were associated to some differences in the effects on the treated parasites. When ring stage parasites were exposed to Ned-19 from 0, 8, or 16 h post-invasion (subcultures 2, 4 and 5), their development did not progress beyond the early trophozoite stages, which accumulated and were observable for the remaining 48 h. When Ned-19 was added later in the asexual cycle, from 24 or 32 h of development (subcultures 6 and 7), the treated early trophozoite stages did not progress in development and accumulated as early schizonts. In subculture 7 a decrease in parasite number was also observed, indicating that incubation of Ned-19 with late trophozoite stages led to parasite cell disruption, unlike it was observed when Ned-19 was incubated with the ring stage parasites.

This experiment indicates that short-term exposure of parasites at the very early phase of the asexual cycle to Ned-19 is not sufficient to block development and, later in the cycle, Ned-19 does not directly affect schizogony and merozoite reinvasion. Ned-19 inhibitory activity is instead concentrated in the trophic part of the asexual cycle and in the development of the trophozoite into the schizont, suggesting that Ned-19 affects distinct cellular processes in the ring and in the trophozoite stages of the asexual parasite.

**Fig. 3:**
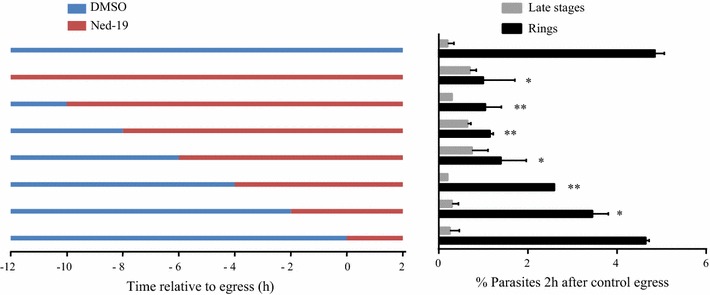
Ned-19 activity on the late phase of the asexual cycle of *P. falciparum*. *Left* asexual *P. falciparum* cultures were synchronized (initial parasitaemia 1.2%) and trophozoites at 32 h post invasion were incubated with DMSO (*blue line*) or 100 μM Ned-19 (*red line*) at the indicated time-points. *Right* parasitaemia and asexual stages of the cultures 2 h after merozoite egress (determined in the DMSO treated culture). N = 2. *p < 0.05; **p < 0.01. *Error bar* range/2

To pinpoint the time at which Ned-19 blocks late asexual development, tightly synchronized late trophozoite stages (32 h after synchronization) were treated with 100 μM Ned-19 at different times spanning a period from 12 h before to 2 h after schizogony, which occurred 44 h after invasion in the untreated control culture (Fig. 3). Parasite morphology and the number of ring stages resulting from successful merozoite reinvasion were measured. The experiment showed that Ned-19 treatment at 12, 10, 8 and 6 h before schizogony resulted in a low number of rings in the next cycle compared to the untreated cultures. This effect was less pronounced, but statistically significant, in the cultures treated 4 and 2 h before schizogony, whereas cultures in which Ned-19 was added at the moment of schizogony showed no difference compared to the DMSO-treated controls. The partial effect observed at the time points immediately preceding schizogony may however be explained by the fact that the 3 h synchronization window used in this experiment would allow some of the parasites to reach the Ned-19 insensitive schizont stage at these time points. This experiment in conclusion confirms that Ned-19 alters the transition from late trophozoite to early schizont and the subsequent parasite maturation into a mature schizont, while it does not affect egress of the merozoites or their ability to reinvade uninfected red blood cells.

To investigate effects of Ned-19 treatment on parasite morphology, late asexual parasites were treated with 62.5 or 125 μM Ned-19 and examined through Giemsa staining and electron microscopy after 6 h of incubation. Examination by light microscopy revealed an aberrant morphology of the late parasite stages, characterized by presence of unstained vacuoles or nuclei with abnormal morphology. These features were present in 48 and 60% of the parasites in the 62.5 μM and the 125 μM treatments, respectively (Additional file 1: Figure S1).

### Ned-19 localizes to acidic compartments in *P. falciparum*

**Fig. 4:**
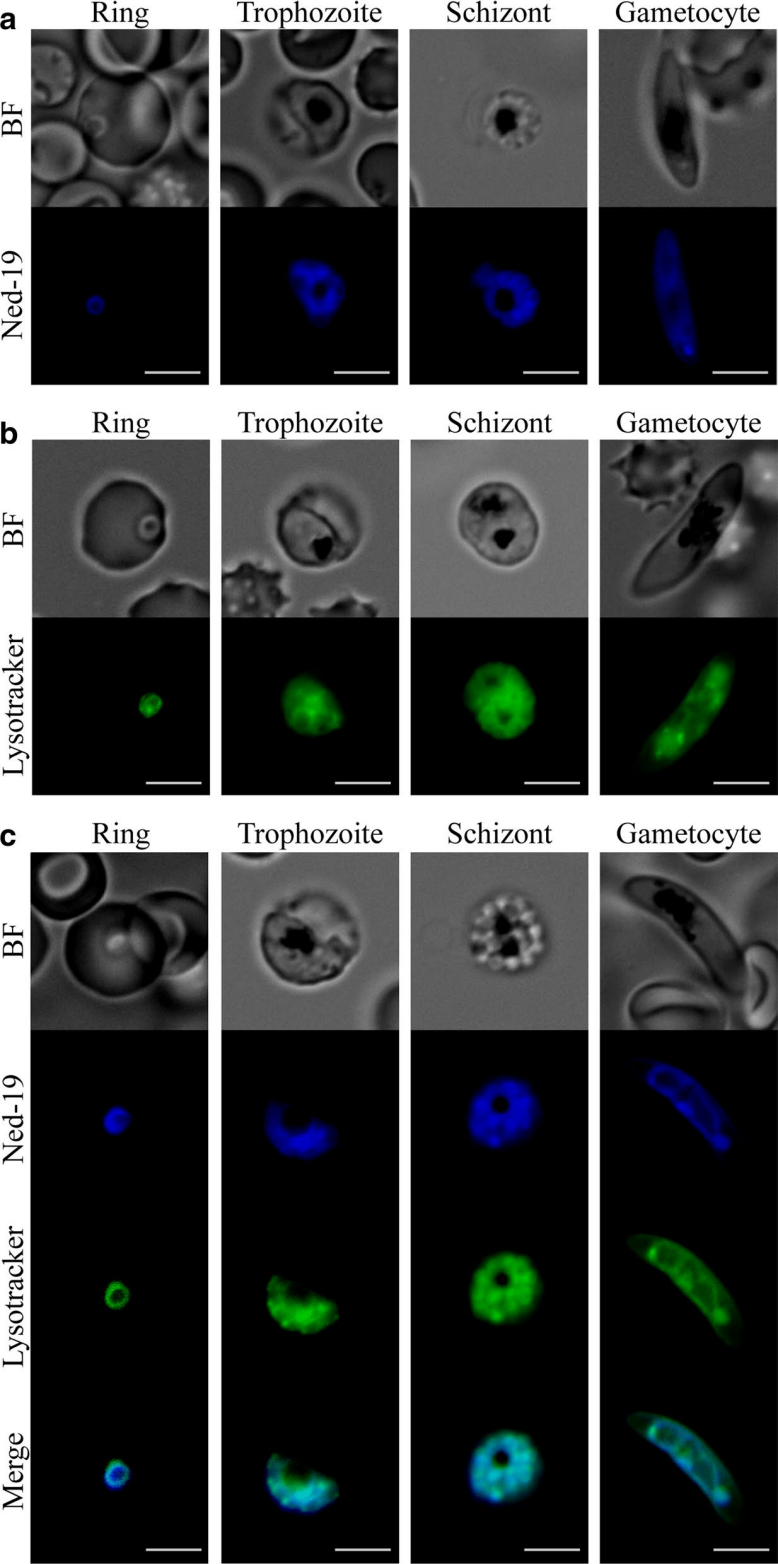
Ned-19 colocalizes with Lysotracker in asexual and sexual parasites. **a** Parasites stained with 200 μM Ned-19. **b** Parasites stained with 1 μM Lysotracker. **c** Parasites stained with 200 μM Ned-19 and 1 μM Lysotracker. *BF* bright field. *Scale bar* 5 μm

Ned-19 is a fluorescent compound and this property has been exploited to label and subcellularly localize the NAADP receptors [25]. To investigate the presence and subcellular localization of putative NAADP receptors in *P. falciparum*, live parasites incubated with 200 μM Ned-19 were examined by fluorescence microscopy. Results showed that Ned-19 fluorescence exhibited a diffuse pattern in the different stages of the asexual cycle and in gametocytes (Fig. 4a).

As Ned-19 localizes to acidic lysosome-like organelles in other cell types, Lysotracker Green DND-26, a dye marking membranes of acidic organelles, was used in co-localization experiments with Ned-19 (Fig. 4b, c). In this experiment a similar subcellular pattern of the Lysotracker and the Ned-19 fluorescence signals in both asexual and sexual parasites was observed, suggesting that Ned-19 accumulates in Lysotracker-positive compartments in *P. falciparum*. This may lead to speculate that putative parasite NAADP receptor(s) may localize in acidic compartments within the parasite, similarly to other organisms [36].

### Ned-19 inhibits spontaneous Ca^2+^ oscillations in early ring and early trophozoite stages

**Fig. 5:**
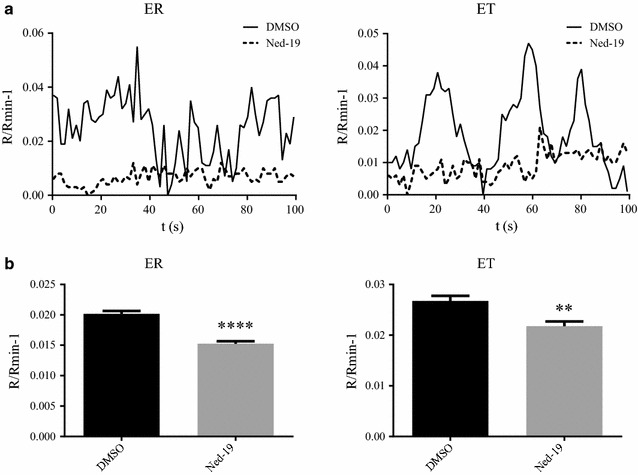
Ned-19 inhibits spontaneous calcium oscillations in *P. falciparum* early rings and early trophozoites. **a** Representative traces of spontaneous calcium oscillations in early rings (ER) and early trophozoites (ET) in the presence of Ned-19 or vehicle DMSO. **b** *Bar charts* showing the average peak height of the spontaneous calcium oscillations in Ned-19 treated early rings (ER) or early trophozoites (ET) compared to controls. **p < 0.01; ****p < 0.0001. *Error bar* SEM

To determine whether Ned-19 affects Ca^2+^ homeostasis in *P. falciparum*, spontaneous oscillations in intracellular Ca^2+^ concentration [6, 37] were measured incubating synchronized early ring and early trophozoite stages loaded with the calcium indicator Fura-2-AM in the presence or absence of 100 μM Ned-19. Oscillations in the intensity of the fluorescent signals were observed in treated and untreated parasites and the respective average peak heights were calculated, with values normalized to the minimum value in each measurement (Fig. 5a). R/Rmin−1 for untreated early rings and trophozoites were 0.0202 (SEM 4.64 × 10^−4^) and 0.0267 (SEM 1.02 × 10^−3^) respectively, whereas in the Ned-19 treated parasites values were 0.01526 (SEM 3.82 × 10^−4^) and 0.02177 (SEM 9.14 × 10^−4^) for early rings and for early trophozoites, respectively. The significantly smaller oscillations observed in the treated ring and trophozoite stages (p < 0.0001 and p = 0.0029, respectively) (Fig. 5b) indicate that spontaneous oscillations in free Ca^2+^ concentration are affected by Ned-19, supporting a specific effect of Ned-19 in *P. falciparum* Ca^2+^ regulation.

## Discussion

This study investigated for the first time the effect of the NAADP antagonist Ned-19 on *P. falciparum* development, revealing that this compound inhibits the parasite asexual cycle and suggesting a potential role of NAADP in regulating *P. falciparum* Ca^2+^ signalling.

While the effects of inositol 1,4,5-bisphosphate (IP3)-induced [22, 23], cyclic ADP-ribose (cADPR) and ATP-induced [38] Ca^2+^ release have been previously described in *P. falciparum* [24] and in *Toxoplasma gondii* [39], the role of NAADP in this process and in parasite physiology has never been investigated in apicomplexan parasites. Moreover, the receptors involved in second messenger-induced Ca^2+^ release are unknown, since the orthologue of both IP3 receptors (IP3Rs) and ryanodine receptors (RyRs) have not been identified and two pore channels (TPCs) NAADP-receptor homologous sequences have not been found in the genome of *P. falciparum* [40]. Results of the present work show that a highly specific inhibitor of NAADP-induced Ca^2+^ release (Ned-19) blocks the transition from early to late trophozoite stages and schizont maturation in *P. falciparum*, supporting the notion that Ca^2+^ signalling plays a crucial role in different stages of *P. falciparum* asexual cycle. This supports previous findings [6] describing a pivotal role of Ca^2+^ signalling in the development of *P. falciparum*. In that study, the stage-specific spontaneous Ca^2+^ oscillations in the intra-erythrocytic stages of *P. falciparum* were inhibited by a specific inhibitor of IP3 (2-APB), resulting in developmental defects and leading to parasite death [6]. Interestingly, in that report and in the present work, the decrease in Ca^2+^ oscillations caused by 2-APB and Ned-19 at the early ring stage was not sufficient to block parasite development, while ability to affect Ca^2+^ oscillations later at the trophozoite stage was in both cases associated to an inhibitory effect on the growth of these parasites. The ability of Ca^2+^ signalling in regulating cellular response in different stages of parasite life cycle relies on a family of Ca^2+^-dependent protein kinases. Among these, PfCDPK1 (calcium-dependent protein kinase-1) is expressed during the intraerythrocytic schizogony and in the sporozoite stage, and it is crucial for the viability of *P. falciparum*. Kato and co-workers identified purkalfamine as an inhibitor of PfCDPK1 able to block parasite development at the late schizont stage [41]. It is known that CDPK1 is crucial also in the sexual life cycle stages of *Plasmodium berghei*, regulating zygote development [42]. Other studies showed that another Ca^2+^-dependent protein kinase, PfCDPK5, controls parasite egress from erythrocytes [43].

These data altogether highlight the potential impact of Ca^2+^ signalling antagonists as new antimalarial drugs able to block different stages of the life cycle of *P. falciparum*. Accordingly, different research groups recently focused their efforts in performing large scale drug screening to identify new compounds able to target Ca^2+^-dependent protein kinases or more generally Ca^2+^-signalling in *Apicomplexa* [44–46]. In this context, the results of this study introduce NAADP signalling as a new potential target for the development of drugs able to impair Ca^2+^ homeostasis in *P. falciparum*.

Spontaneous Ca^2+^ oscillations have been poorly investigated in *P. falciparum*. It has been shown that a specific inhibitor of IP3 (2-APB) was able to block Ca^2+^ oscillations in ring and trophozoite stages [6], while a selective melatonin receptor antagonist, luzindole, was able to inhibit spontaneous Ca^2+^ oscillations mainly in the ring stages of the parasite [37]. The result that Ned-19 affects Ca^2+^ oscillations in early rings and trophozoites suggests that both NAADP and IP3 are involved in this process. This result is consistent with the observations that NAADP induces Ca^2+^ oscillations in different eukaryotic cell types, from sea urchin eggs [19] to pancreatic acinar cells [47–49], through a two-pool mechanism involving both endoplasmic reticulum-independent NAADP-sensitive stores and the endoplasmic reticulum IP3- and cADPR-sensitive stores [50, 51]. In this model, NAADP is crucial in priming Ca^2+^-induced Ca^2+^-Release from endoplasmic reticulum (CICR), through a mechanism of overloading and spontaneous Ca^2+^ release from these stores [50].

Besides using Ned-19 as a specific inhibitor of NAADP signalling, this compound was used to fluorescently tag the NAADP receptor in living cells [25]. These experiments showed the co-localization of the Ned-19 and the Lysotracker fluorescent signals in different stages of the life cycle of *P. falciparum*, suggesting that yet to be identified NAADP-receptors may localize on acidic compartments also in *P. falciparum* and possibly in *Apicomplexa*, similarly to several mammalian cell types [18, 20]. As however different acidic organelles are labelled by Lysotracker dye in *P. falciparum* (acidocalcisomes, digestive vacuole and lysosomes), further experiments are needed to identity the Ned-19 positive organelles.

## Conclusion

The ability of the specific NAADP inhibitor Ned-19 to block *P. falciparum* life cycle progression from the trophozoite to the late asexual stages and to affect spontaneous oscillations in the parasite intracellular Ca^2+^ concentration constitute the first evidence to suggest the presence of the regulatory molecule NAADP and its role in Ca^2+^ homeostasis in the malaria parasites and more generally in *Apicomplexa*. The lack of predicted orthologue NAADP receptors in the *P. falciparum* genome suggests that such putative receptors and the NAADP mediated regulatory mechanism(s) in the malaria parasites may considerably differ from those described in distant organisms. This raises the possibility that these yet to be explored cellular mechanism(s) may be targeted with a high selectivity.

## Additional files

**Additional file 1: Figure S1.** Ned-19 induces morphological changes in the structure of late asexual stages of *P. falciparum*. Late stages parasites were incubated for 6 h in the presence or absence of 125 μM Ned-19 and processed to examine their morphology. A) Samples were Giemsa stained and examined for conspicuous morphological alterations. Representative untreated and treated parasites are shown. Scale bar: 5 μm. B) Electron Micrographs showing representative DMSO and Ned-19-treated parasites. R: Rhoptry. N: Nucleus. Scale bar: 0.5 μm.

**Additional file 2: Figure S2.** Representative examples of asexual parasites. ER: Early rings; LR: Late rings; ET: Early trophozoites; LT: Late trophozoites; ES: Early schizonts; LS: Late schizonts. Scale bar: 5 μm.

## Abbreviations

Ca^2+^: calcium
ATPase: adenosine triphosphatase
PPase: pyrophosphatase
SERCA: sarco/endoplasmic reticulum Ca^2+^-ATPase
IP3: inositol 1,4,5-bisphosphate
cADPR: cyclic ADP-ribose
NAADP: nicotinic acid adenine dinucleotide phosphate
TPCs: two pore channels
vWF: von Willebrand factor
ET-1: endothelin-1
DMSO: dimethyl sulfoxide
ROI: region of interest
SEM: standard error mean
IP3R: IP3 receptor
RyR: ryanodine receptor
PfCDPK1: calcium-dependent protein kinase-1
CICR: Ca^2+^-induced Ca^2+^-release
ER: early rings
LR: late rings
ET: early trophozoites
LT: late trophozoites
ES: early schizonts
LS: late schizonts
