# Supplementary figures and images for: Ned-19 inhibition of parasite growth and multiplication suggests a role for NAADP mediated signalling in the asexual development of Plasmodium falciparum
Source: Malar J. 2017 Sep 12;16:366. doi: 10.1186/s12936-017-2013-7 (PMC5596470; doi:10.1186/s12936-017-2013-7)

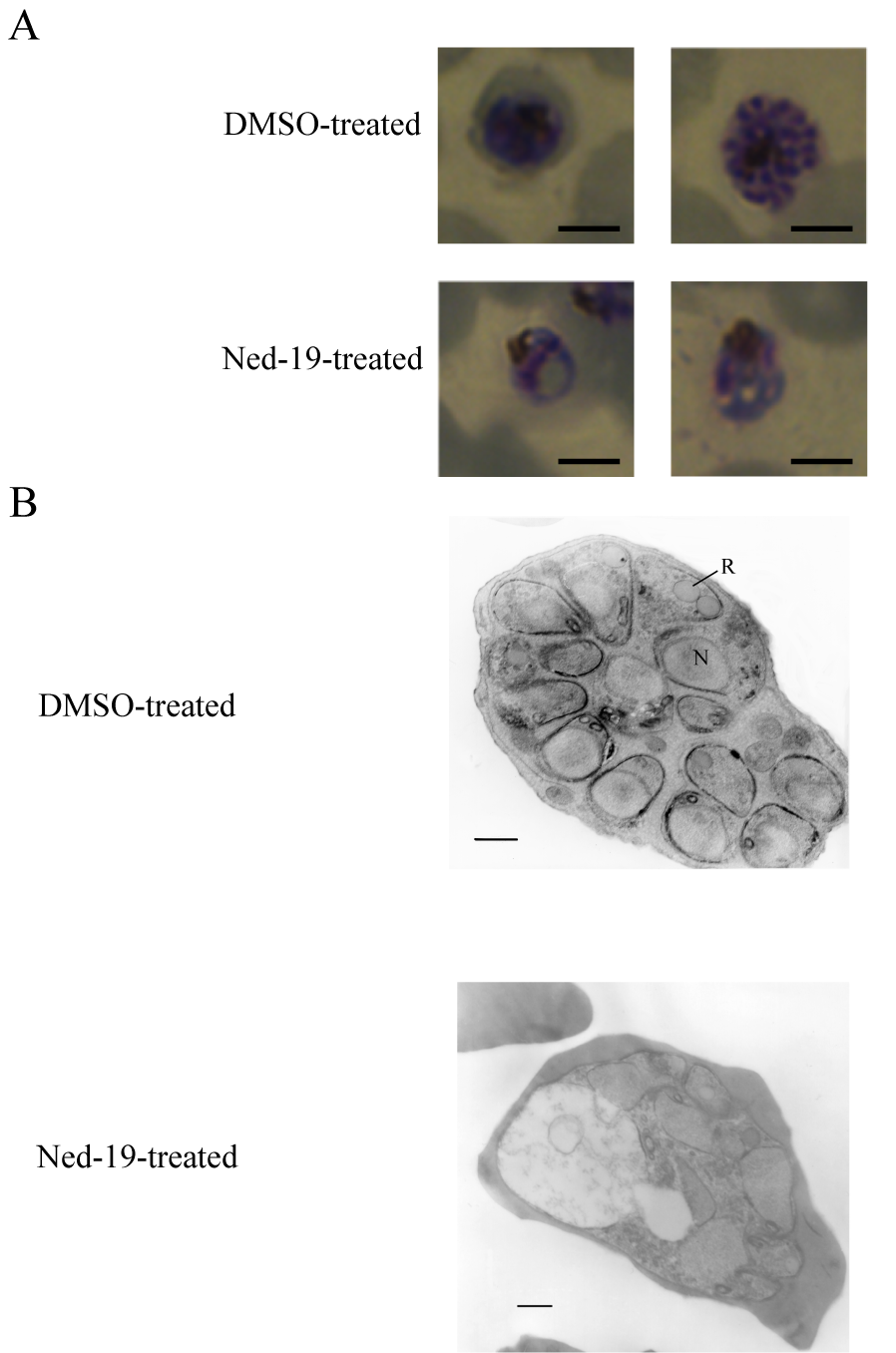

Supplement: Supplementary file 1 — Additional file 1: Figure S1. Ned-19 induces morphological changes in the structure of late asexual stages of P. falciparum. Late stages parasites were incubated for 6 h in the presence or absence of 125 μM Ned-19 and processed to examine their morphology. A) Samples were Giemsa stained and examined for conspicuous morphological alterations. Representative untreated and treated parasites are shown. Scale bar: 5 μm. B) Electron Micrographs showing representative DMSO and Ned-19-treated parasites. R: Rhoptry. N: Nucleus. Scale bar: 0.5 μm. [file 12936_2017_2013_MOESM1_ESM.tif]

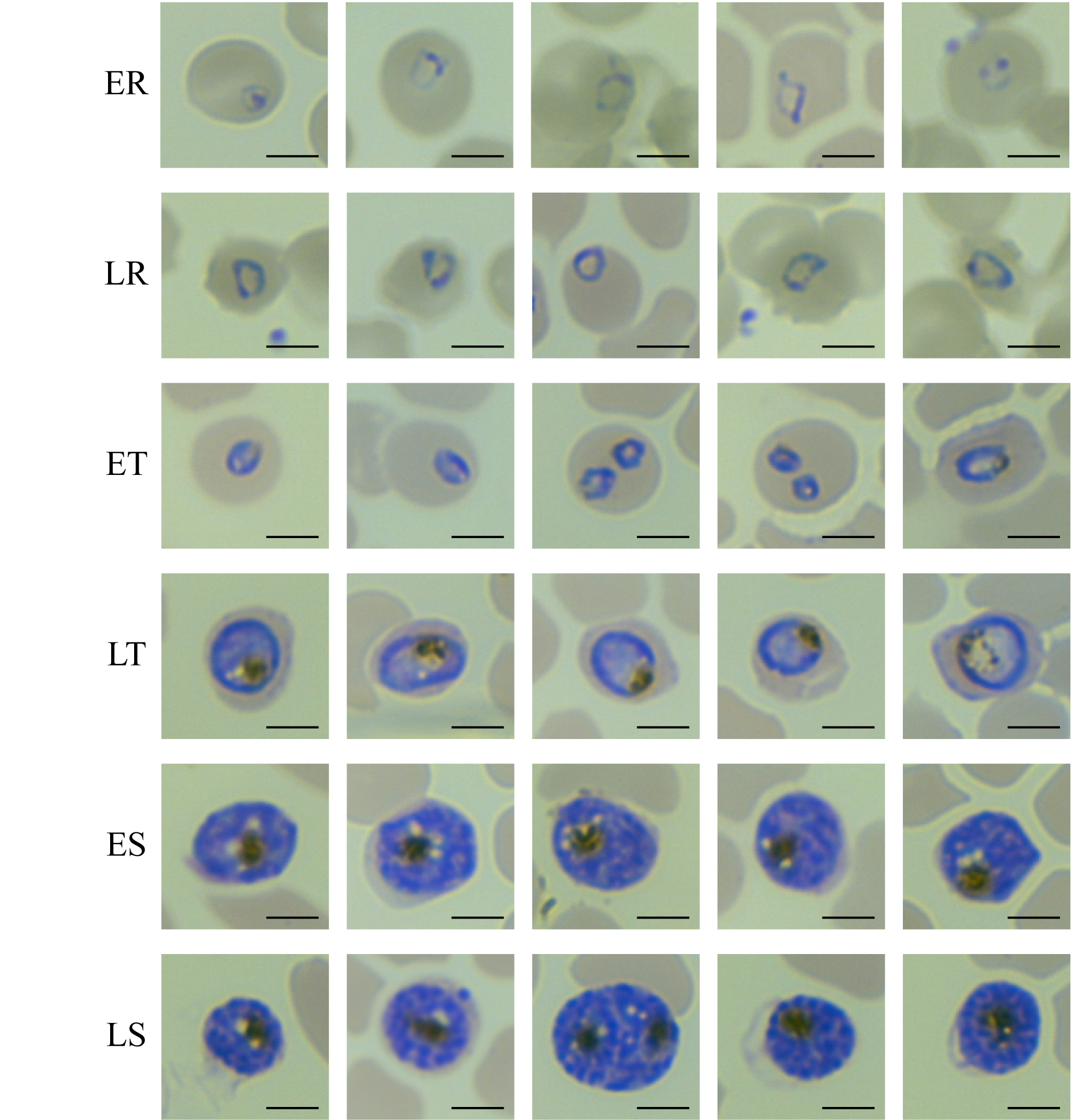

Supplement: Supplementary file 2 — Additional file 2: Figure S2. Representative examples of asexual parasites. ER: Early rings; LR: Late rings; ET: Early trophozoites; LT: Late trophozoites; ES: Early schizonts; LS: Late schizonts. Scale bar: 5 μm. [file 12936_2017_2013_MOESM2_ESM.tif]
